# Numerical discrimination in Danionella

**DOI:** 10.1016/j.isci.2025.113667

**Published:** 2025-09-30

**Authors:** Mirko Zanon, Scott E. Fraser, Giorgio Vallortigara

**Affiliations:** 1 Centre for Mind/Brain Sciences, University of Trento, Rovereto, Italy; 2 Translational Imaging Center, Michelson Center for Convergent Bioscience, University of Southern California, Los Angeles, CA, USA; 3 Molecular and Computational Biology, University of Southern California, Los Angeles, CA, USA; 4 Quantitative and Computational Biology, University of Southern California, Los Angeles, CA, USA

**Keywords:** Neuroscience, Cognitive neuroscience, Techniques in neuroscience

## Abstract

The transparent teleost *Danionella cerebrum* offers unique advantages for neuroscience research, including small size, lifelong cranial transparency, and a simplified brain. Despite its suitability for imaging and neural circuit studies, its cognitive abilities remain largely unexplored. Here, we show that *Danionella* can discriminate between quantities. Using a habituation/dishabituation paradigm over six trials across five days, fish were exposed to dot arrays of constant numerosity (e.g., 3 dots), while geometric visual properties were systematically varied to ensure responses were driven by actual numerical information. In the final trial, a different numerosity (e.g., 9 dots) was presented. Fish showed significant dishabituation, spending more time near the numerically altered stimulus, indicating discrimination between 3 and 9 elements. These findings contribute to our understanding of *Danionella*’s cognitive capacities and support its broader use in research, particularly for investigating the neural basis of cognition at high resolution, given its exceptional biological features.

## Highlights

• *Danionella cerebrum* demonstrates numerical discrimination
• Using a habituation/dishabituation paradigm, *Danionella* distinguishes between 3 and 9 dots
• Findings support *Danionella* as a model for studying the neural basis of numerical cognition

## Graphical abstract

**Figure:**
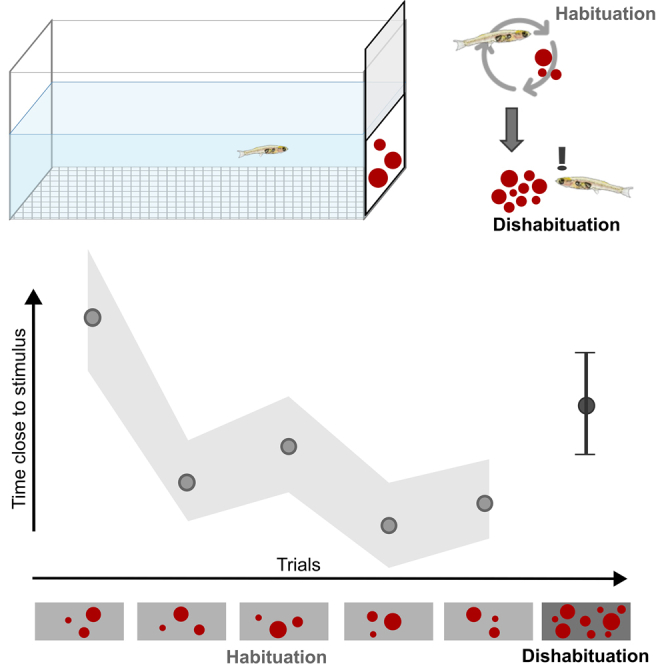

## Introduction

The emergence of *Danionella cerebrum* as a model organism in neuroscience is opening exciting opportunities for studying neural mechanisms underlying vertebrate cognition.^1^^,^^2^^,^^3^^,^^4^^,^^5^^,^^6^^,^^7^

A member of the cyprinid family, *Danionella cerebrum* is a small, transparent teleost that shares phylogenetic proximity with the zebrafish (*Danio rerio*) but differs markedly in traits relevant to neuroscience research. Adult *Danionella* are significantly smaller (∼1 cm in length) than zebrafish (∼3–4 cm), and uniquely retain cranial transparency throughout their lifespan, allowing non-invasive, long-term imaging of neural activity in the adult brain.^1^^,^^5^^,^^8^ Their reduced pigmentation, compact body size, and smaller brain enhance imaging resolution and simplify whole-brain and circuit-level analyses. Both species are genetically accessible, but *Danionella*’s anatomical features make it particularly well suited for high-resolution techniques such as *in vivo* calcium imaging and optogenetics in adults. These characteristics position *Danionella cerebrum* as a powerful complementary model for adult systems neuroscience.

Although behavioral studies in *Danionella* are still limited, its evolutionary closeness to zebrafish suggests it may possess comparable cognitive abilities, an assumption that remains to be empirically tested for many different domains. While extensive research has documented zebrafish behavior and cognition,^9^ including learning,^10^ memory,^11^ and numerical abilities,^12^^,^^13^^,^^14^ analogous behavioral data for *Danionella cerebrum* remain largely absent. Only a few behavioral studies were performed, mainly focusing on visual spatial navigation,^15^ locomotor activity,^16^ schooling^8^ and vocal behavior.^17^^,^^18^^,^^19^^,^^20^

The gap still represents a critical opportunity to explore the potential of *Danionella* as a model organism for studying cognitive processes (despite the current evidence for some cognitive abilities mentioned before), linking behavior to neural mechanisms in a way that is challenging with other vertebrates.

In this study, we address this gap by investigating numerical cognition in this fish. Numerical cognition (i.e., the ability to perceive, process, and discriminate quantities) is an essential skill for many animal species. It supports critical behaviors, such as foraging, predator avoidance, and social decision-making, and is thought to be widespread across taxa.^21^^,^^22^^,^^23^^,^^24^ Teleost fish, including zebrafish and guppies, have demonstrated the capacity to process numerical information,^12^ typically using paradigms that isolate numerical perception from other visual properties, such as area or contour length.^25^^,^^26^^,^^27^ For example, guppies have been observed to distinguish between different numbers of foraging tasks,^28^ while zebrafish, already at larval stage, prefer the largest amount of familiar environmental elements such as vertical bars.^29^

**Figure 1:**
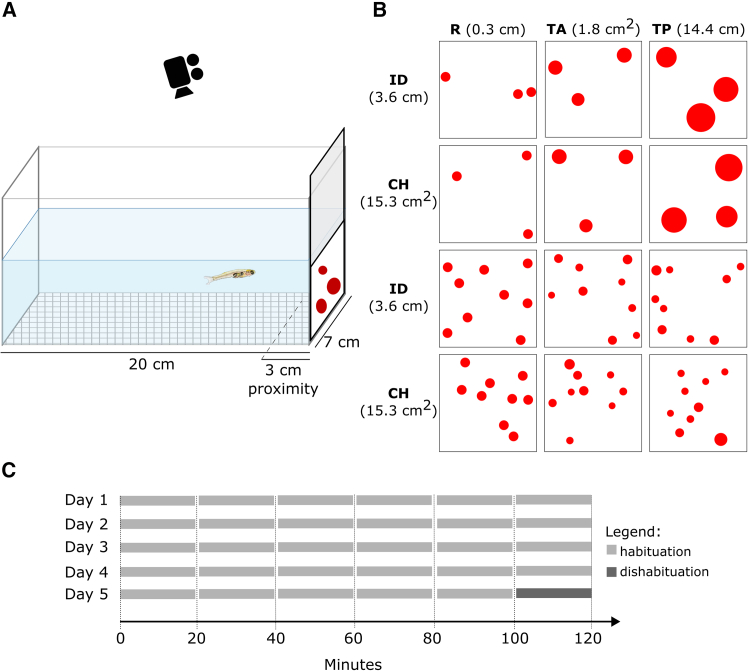
Experimental setup and stimuli (A) Schematic of the experimental corridor. The proximity area, arbitrarily defined as the zone within 3 cm of the stimulus, represents the region where fish are considered to be investigating the stimulus. (B) Example of experimental numerical stimuli (3 or 9 dots), controlled for physical variables such as dot radius (R), total dot area (TA), total perimeter (TP), average inter-dot distance (ID), and overall convex hull (CH). (C) Schematic of the experimental pipeline: Each day included six consecutive 20-min habituation trials. On the final day (Day 5), a new numerosity was presented during the last trial (trial 6) to evaluate dishabituation.

To investigate numerical abilities in *Danionella cerebrum*, we employed a habituation/dishabituation paradigm, a well-established method in neuroscience for testing non-verbal organisms (in particular largely used with infants^30^^,^^31^). It was recently introduced in zebrafish for studying numerical cognition.^25^^,^^26^ This paradigm leverages an organism’s natural tendency to habituate to repeated stimuli and subsequently show heightened responses to novel ones. In this context, the habituation occurs with a prolonged exposition of *Danionella* to a specific numerosity (gradually occurring across 6 consecutive trials of 20 min of stimuli presentation for 5 days), disrupted by the presentation of a novel numerosity in the very last trial (trial 6 of day 5) which should elicit dishabituation, if perceived as substantially different from previous stimuli. More specifically, habituation was tested by presenting individual fish with repeated stimuli of a fixed numerosity (e.g., 3 dots -or 9 dots-), systematically varying continuous properties such as total perimeter, total area, inter-distance and convex hull to control for non-numerical factors. Dishabituation was then tested by introducing a novel numerosity (e.g., 9 dots -or 3 dots, respectively-; consistent with previous geometrical factors) in the final trial, with the behavioral response (e.g., time spent near the numerosity stimulus) indicating numerical discrimination. See Figure 1 for a schematic of the setup and experimental paradigm, as well as for examples of stimuli employed.

Our focus on a basic numerical discrimination task, comparing 3 versus 9 dots, reflects a strategic approach to assess *Danionella cerebrum*’s numerical abilities. These numerosities are convenient for distinguishing between small and large quantities, while being far apart enough (in terms of ratio) to ensure robust behavioral responses. Similar tasks have been successfully used with zebrafish,^25^^,^^26^ providing a comparative framework for interpreting *Danionella*’s performance.

This study investigates numerical cognition in *Danionella cerebrum*, assessing its ability to distinguish between different numerical quantities as part of a broader effort to characterize its cognitive profile. In doing so, we lay the groundwork for future research linking numerical processing to neural activity, taking advantage of *Danionella*’s unique traits, such as lifelong cranial transparency and suitability for high-resolution imaging. Beyond species-specific insights, our findings contribute to a comparative understanding of numerical cognition across vertebrates, offering perspective on the evolutionary and functional relevance of this cognitive ability in teleost fish. Crucially, we emphasize that robust behavioral validation is essential before exploring the neural correlates of cognition. Our contribution is therefore 2-fold: it provides empirical behavioral data on numerical discrimination in *Danionella*, and it establishes a practical, flexible paradigm that can be adapted for future cognitive and neurobiological investigations in this emerging model organism.

## Results

### Evidence for habituation in *Danionella*

**Figure 2:**
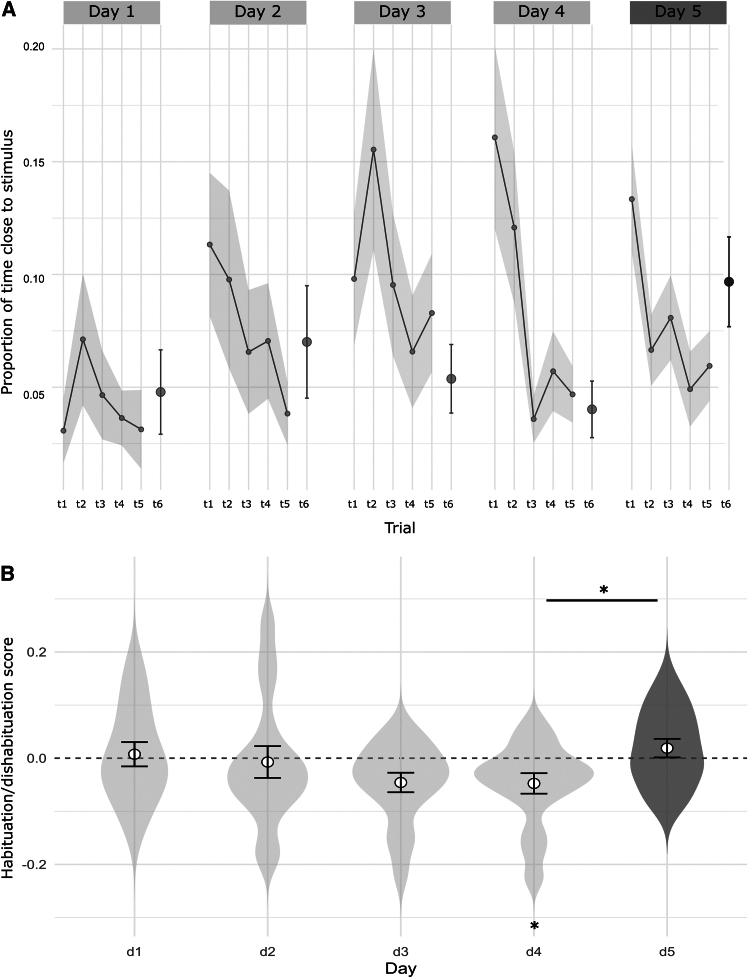
Numerical habituation/dishabituation results (A) Habituation curves across six trials over the five experimental days. Values represent the proportion of time spent near the stimulus during the first 180 s of each trial. Trial 6 (t6) corresponds to the reference trials to compute the habituation/dishabituation score (and for this reason depicted separately as a dot). Data are represented as mean ± SEM; *N* = 16 subjects. (B) Habituation/dishabituation scores for the five experimental days, calculated as the difference between the proportion of time spent near the stimulus in the final daily trial and the average of habituation trials 1 to 5. Data are represented as mean ± SEM overlaid on violin plots; *N* = 16 subjects. (∗ = permutation ANOVA *p* < 0.05).

The habituation/dishabituation results for the numerical comparison of 3 versus 9 in *Danionella cerebrum* are summarized in Figure 2, where the whole habituation curves (evaluated by the proportion of time spent close to the stimulus) across the 5 experimental days (Figure 2A) and the habituation/dishabituation scores (Figure 2B) are reported. The habituation/dishabituation score was calculated as the difference between the proportion of time spent near the stimulus in the last (sixth) daily trial and the average proportion of time spent near the stimulus during the first five trials. This summary metric captures the performance of the paradigm: negative scores indicate reduced proximity to the stimulus in the final trial, reflecting habituation, whereas positive scores indicate renewed interest in the stimulus, serving as a proxy for dishabituation.

Across days 1–4, *Danionella* required time to familiarize themselves with the experimental setup and stimuli, gradually developing a stable habituation response, as evidenced by a daily decrease in time spent near the familiar stimulus across trials, together with a consistent decrease in the habituation score across days. The fact that actual habituation occurred by the last trial of day 4 is also confirmed by a significant negative difference from a zero score, for the habituation score at day 4 (t(15) = −2.5, *p* = 0.03; Cohen’s *d* = −0.6).

### Numerical discrimination (3 vs. 9) revealed by dishabituation in *Danionella*

For the final discrimination assessment, day 5 serves as the critical test day when dishabituation occurs, marked by the introduction of a novel numerosity in the final trial. To evaluate a relative effect to the previous habituation, we statistically tested dishabituation, analyzing the difference between the habituation/dishabituation scores on day 4 (the last full day of habituation) and day 5 (the dishabituation day).

Moreover, we considered the two types of experimental groups (“*group type*” factor): habituation with 3 dots and dishabituation with 9 dots, or habituation with 9 dots and dishabituation with 3 dots. A significant effect of the “*day*” factor was observed (F = 5.37, permutation ANOVA with 5000 permutations, *p* = 0.03; effect size partial η^2^ = 0.28), with no significant effect of the “*group type*” factor (habituation with 3 dots or with 9 dots; F = 2.6, permutation ANOVA with 5000 permutations, *p* = 0.13; effect size partial η^2^ = 0.10) or interaction (F = 0.34, permutation ANOVA with 5000 permutations, *p* = 0.57; effect size partial η^2^ = 0.02). The significant difference between habituation and dishabituation days confirm that a final numerical discrimination occurs, which is not dependent on the type of habituation (if with 3 or 9 dots).

### Habituation curves vary with control group (smaller vs. larger numerosity)

**Figure 3:**
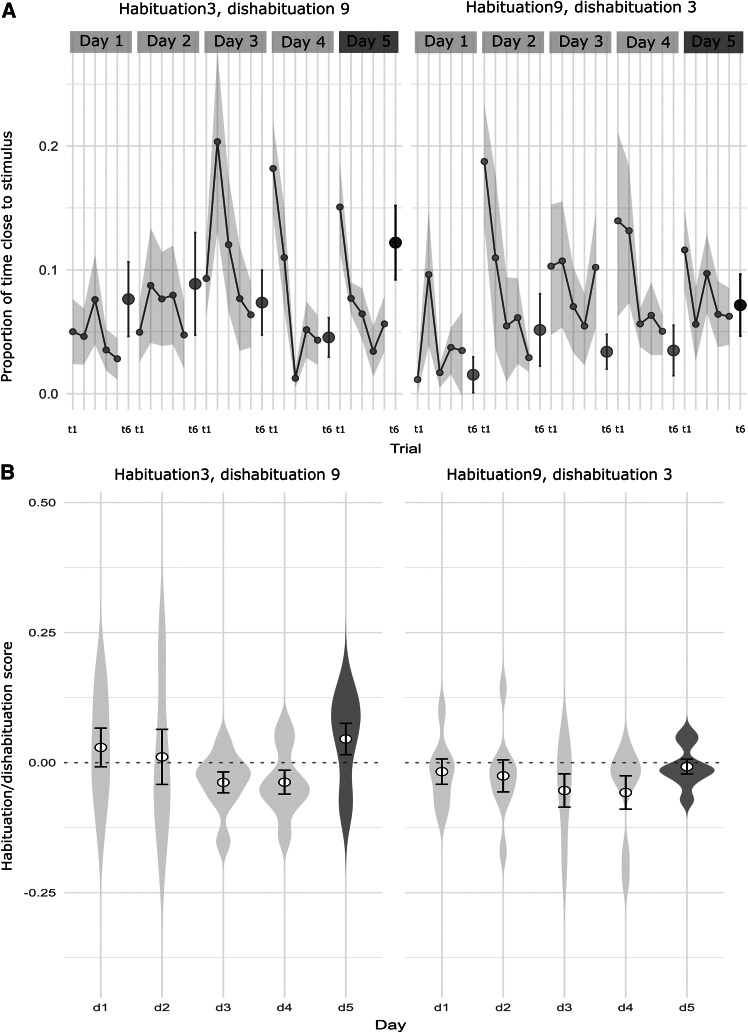
Scores dived by group (habituated with 3 or with 9 dots) (A) Habituation curves across six trials over the five experimental days by experimental group. Values represent the proportion of time spent near the stimulus during the first 180 s of each trial. Trial 6 (t6) corresponds to the reference trials to compute the habituation/dishabituation score (and for this reason is depicted separately as a dot). Data are represented as mean ± SEM; *N* = 8 + 8 subjects. (B) Habituation/dishabituation scores for the five experimental days by experimental group, calculated as the difference between the proportion of time spent near the stimulus in the final daily trial and the average of habituation trials 1 to 5. Data are represented as mean ± SEM overlaid on violin plots; *N* = 8 + 8 subjects.

Despite this absence of significance between the dishabituation trends of the two groups (habituated with 3 or 9 dots), it could be still interesting to report from a behavioral descriptive point of view how habituation occurs within them: this is shown in Figure 3. While the decreasing trend (across day 1–4) followed by an increase at day 5 is analogous (as resulted in *day* factor significance of the previous test with absence of *group* significance, with *n* = 8 per group), this dishabituation is happening with different baseline habituation behavior. For example, by looking at the first two days (in Figure 3) it can be noticed how fish showed a weaker initial habituation to the smallest number of items (overall score average for days 1–2, habituation with 3: +0.02 ± 0.03; habituation with 9: −0.02 ± 0.02).

## Discussion

This study demonstrates that *Danionella cerebrum* possesses numerical discrimination abilities, as evidenced by its ability to distinguish between different numerosities (3 vs. 9) using a habituation/dishabituation paradigm. These findings not only contribute to the growing literature on numerical cognition in teleost fish but also highlight the behavioral flexibility of *Danionella* and suggest that numerical processing may be more widespread among teleosts than previously recognized. Furthermore, the unique traits of *Danionella*, including its cranial transparency, small size, and accessibility to advanced imaging technologies, position it as a highly promising model organism for bridging behavioral studies of cognition with detailed neural circuit analyses.

Overall, our results demonstrate a clear habituation/dishabituation pattern in *Danionella* when exposed to numerical comparisons between 3 and 9 dots, irrespective of the stimulus type used for habituation or dishabituation (3 to habituate and 9 to dishabituate, or vice versa). This conclusion is further strengthened by the design of our stimuli, which systematically varied confounding physical variables such as dot radius, total area, total perimeter, mean inter-dot distance, and convex hull. By controlling and varying these factors, we ensured that the habituation was driven by numerical information rather than by non-numerical visual cues. These findings not only confirm the feasibility of this paradigm for studying numerical cognition in *Danionella* but also highlight their ability to process numerical differences in a controlled experimental setting.

The results of the analysis revealed a significant habituation effect at day 4 (average dishabituation score of −0.047, 95% CI: −0.089,−0.006). This suggests a strong degree of habituation by the last trial of the day, as negative scores correspond to a greater reduction in response. The effect size η^2^, as measured by Cohen’s d = −0.6, represents a medium-to-large effect, highlighting this substantial reduction in response on day 4. An *a posteriori* power analysis (considering a match pair comparison with 16 fish, alpha level at 0.05 and d = 0.6) indicates an achieved power of 0.74.

While the main analysis focuses on how preference in the last trial of the series changes over days, other behavioral patterns emerge from the time fish spent near the stimuli (Figure 2A). For instance, time near the stimulus tends to increase after the first couple of days: this early-session exploratory behavior may reflect general arousal or spontaneous dishabituation, which grows as fish become more familiar with the setup. Consistently, we observed elevated exploration during the first trial of each day, likely due to overnight dishabituation or novelty effects. Nonetheless, for our experimental questions, the most informative measure remains the relative change in behavior by the final trial (trial 6), when baseline arousal has typically subsided. On habituation days, when the same numerosity was presented across trials, exploration reliably declined by trial 6, regardless of the initial level of interest. In contrast, on the test day involving a change in numerosity during trial 6, we observed an increase in exploration, supporting the interpretation of a genuine dishabituation response. A possible factor to explain the observed behavioral changes across days could be familiarization with the setup or with the repeated presentation of stimuli. Indeed, increased time spent near the stimuli across days could reflect growing comfort with the apparatus or reduced stress, while declines within a session may indicate habituation to repeated exposure. Our design helps to disentangle these possibilities in two key ways. First, by comparing habituation days (constant numerosity) with the test day (change in numerosity at trial 6), we can directly contrast responses to stimulus repetition versus stimulus change. The consistent decline in exploration within habituation days suggests that familiarization alone cannot account for the renewed response on the test day. Second, the fact that the increase is specifically tied to the numerosity change in the final trial, rather than to the mere progression of trials or days, strengthens the interpretation of a dishabituation effect.

By comparing the score on day 4 and final day 5 we also found a significant difference between these two days, confirming the dishabituation occurrence (factor “*day*”: *p* = 0.03, permutation ANOVA). Specifically, the partial eta-squared of 28% for the dishabituation demonstrate a large effect size, underscoring the fish’s ability to detect changes in numerosity from the habituated stimulus (3 or 9 dots) to the novel one (9 or 3 dots, respectively). Furthermore, because the physical properties of the stimuli (e.g., area, perimeter, inter-dot distances, convex-hull) were controlled, this response represents a pure numerical effect rather than a reaction to low-level visual features.

The factor “*group type*” (i.e., whether the fish was habituated to 3 and dishabituated to 9, or vice versa) did not yield significant results in the dishabituation phases, with a partial η^2^ of 0.10. These small effect sizes indicate that the order of the numerosity presentation did not influence the dishabituation process. This finding suggests that the observed effects are consistent regardless of the specific numerosity used during habituation and dishabituation. It is interesting to notice that also previous work in zebrafish^25^^,^^26^ with a similar task of habituation/dishabituation to numerosity showed no dishabituation difference between groups habituated with smaller or larger number of dots, consistently with our results. Some dissimilarities in the habituation process can still be observed (Figure 3), with lower scores for the group habituated with 9 dots and tested with 3 dots, in particular during the first habituation days. This may be due to the larger number of elements, which could generally cause the fish to stay relatively farther away from them, while 3 dots could induce more confidence to fish which persist in their stimuli investigation for longer time (with a consequent weaker initial habituation). This difference is anyway not influencing the overall habituation curve and dishabituation effect (increase of time close to stimulus in trial 6 of day 5 with respect to trials 1–5), which is consistent across fish (Figure S1).

Additionally, the amount of time *Danionella* spent near the stimuli could be noteworthy. The proportion of time ranged between 0.05 and 0.15, corresponding to approximately 7–21 s out of the 180 s analyzed, indicating that these fish generally spent a large amount of time away from the stimuli. This observation may reflect a general behavioral trait of *Danionella*. Consistent with communications with researchers using these animals, *Danionella* are often reported as highly timid and challenging to engage in behavioral experiments, thus we believe the absolute time values reflect the fact that these fish prefer to keep a certain distance from novel stimuli, in particular on the first days of acclimation. However, results from zebrafish^25^^,^^26^ show that, in a similar paradigm, these fish spent about 10–15% of their time near novel numerical stimuli during a 30-s investigation period, making our scores (5–15%) comparable.

In summary, this study provides robust evidence for both habituation and dishabituation in *Danionella cerebrum*, demonstrating its ability to discriminate between numerical quantities. Beyond the specific findings, the implementation of a functional habituation/dishabituation paradigm represents an important step toward establishing *Danionella* as a viable model for behavioral research. Given the species’ unique biological features, such as cranial transparency and reduced brain dimension, this behavioral framework offers a critical foundation for future studies aiming to link cognition with neural mechanisms at high resolution. Taken together, these results position *Danionella cerebrum* as a promising system for integrative research in cognitive neuroscience.

### Limitations of the study

While we acknowledge that the current dataset is limited and preliminary, the conclusions should be interpreted with caution and within their proper context of validity. As a potential future improvement, additional experiments could be conducted with a larger sample size to further validate the findings and strengthen the generalizability of the results. In the present study, the range of numerical comparisons was limited by practical constraints, including time and subject availability. We focused on two numerosities (3 and 9) chosen for their simplicity and a high 1:3 ratio, which has been shown in previous studies to be reliably discriminated by young zebrafish larvae.^25^^,^^29^ Given the lack of previous evidence on numerical abilities in *Danionella cerebrum*, our aim was to adopt a ratio that was likely to yield measurable effects. Despite this limited range, the current findings clearly demonstrate that *Danionella* can discriminate numerical information using a habituation/dishabituation paradigm, thus establishing a solid foundation for future research.

Given that our *a priori* power analysis was based on a relatively large expected effect size, we acknowledge this could limit the interpretability of potential null results due to reduced sensitivity to smaller effects. This limitation is particularly relevant when interpreting the absence of differences between experimental groups (habituated with 3 or 9 dots, see Figure 3), where fewer subjects per group may have further reduced statistical power. However, the main overall finding aligned with our expectations, and the achieved power (0.74) supports the reliability of the observed positive result.

A potential limitation of the study lies in the use of a single dishabituation stimulus per fish, which may restrict the ability to fully disentangle numerosity from other geometric features during the test phase. However, this concern is mitigated by two key aspects of our design. First, the control for physical variables is inherently built into the habituation phase: fish were exposed to a wide range of geometric configurations and spatial layouts where only numerosity was held constant. As such, features such as total area, perimeter, or density varied systematically across trials, preventing habituation to any specific non-numerical cue. In this sense, successful habituation itself functions as a direct control for these variables. Second, the dishabituation stimulus was presented in a between-subject design, introducing variability across individuals in terms of low-level geometric properties. This additional variation further reduces the possibility that responses were driven by any specific uncontrolled cue. While the inclusion of multiple dishabituation sessions could offer even more targeted dissociation of geometric factors, we believe our design provides a robust and internally consistent framework for attributing observed responses to numerical changes, while keeping the habituation/dishabituation paradigm clean.

Future research should expand the range of tested numerosities to not only explore whether *Danionella* can generalize across ratios but also engage different cognitive mechanisms. Although the current study did not aim to distinguish between the approximate number system (ANS), which supports estimation, and the object tracking system (OTS), which operates in the subitizing range, future work may explore these distinctions. Additionally, combining behavioral paradigms with advanced techniques such as *in vivo* calcium imaging could provide insight into how numerical information is encoded and processed at the circuit level.

An additional limitation is the sex influence on this task. While we did not consider it here (due to the too limited number of available subjects), future works can investigate if some sex differences are relevant.

Finally, while this study establishes that *Danionella* is capable of numerical discrimination under controlled experimental conditions, it remains to be tested whether such abilities are used in ecologically relevant contexts. Demonstrating a cognitive skill in the lab does not necessarily imply ecological utility.^15^ Future studies might therefore adopt paradigms with higher ecological validity, such as shoal choice or foraging tasks, to investigate whether numerical competence in *Danionella* contributes to individual fitness and may have been shaped by natural selection.^32^

## Resource availability

### Lead contact

Further information and requests should be directed to and will be fulfilled by the Lead Contact, Mirko Zanon (mirko.zanon@unitn.it).

### Materials availability

This study did not generate new unique reagents nor new animal lines.

### Data and code availability

• Data: Data (time spent by fish in the experimental arena sectors) are available in Figshare repository: https://doi.org/10.6084/m9.figshare.28271237.
• Code: Code used for the analysis of this work is available in Figshare repository: https://doi.org/10.6084/m9.figshare.28271237.
• Additional information: Any additional information can be required to lead contact.

## Acknowledgments

We thank Benjamin Judkewitz (Einstein Center for Neurosciences, Charité Universitätsmedizin Berlin) for providing the fish line, Matthew Lovett-Barron (School of Biological Sciences, University of California San Diego) and Thai V. Truong (Translational Imaging Center, University of Southern California) for their contributions. We are grateful to all of them for the insightful scientific discussions regarding the implementation of this animal model. This project was funded by ERC European Union's Horizon 2020 research and innovation program Grant agreement 833504 – SPANUMBRA to GV; by FARE–Ricerca in Italia: Framework per l'Attrazione ed il Rafforzamento delle Eccellenze per la ricerca in Italia, III edizione, project “NUMBRISH–The neurobiology of numerical cognition: searching for a molecular genetic signature in the zebrafish brain” Prot. R20YL9WN9N to GV; by PRIN -Progetti di rilevante Interesse Nazionale 2022- PNRR Grant Agreement P2022TKY7B: “The emergence of proto-arithmetic abilities with empty and non-empty sets” to G.V.

## Author contributions

Conceptualization: MZ and GV.

Setup implementation: MZ.

Methodology: MZ and GV.

Investigation: MZ, SF, and GV.

Visualization: MZ.

Funding acquisition: GV.

Supervision: SF and GV.

Writing – original draft: MZ.

All authors reviewed and edited the final version of the article.

## Declaration of interests

The authors declare no competing interests.

## Declaration of generative AI and AI-assisted technologies in the writing process

During the preparation of this work, the authors used ChatGPT exclusively to improve English and readability in some sentences. After using this tool, the authors reviewed and edited the content as needed and take full responsibility for the content of the publication.

## STAR★Methods

### Key resources table

**Table:** 

REAGENT or RESOURCE	SOURCE	IDENTIFIER
**Deposited data**		

Data for time spent by fish close to stimuli for all trials	This paper	https://doi.org/10.6084/m9.figshare.28271237
R script for statistical data analysis	This paper	https://doi.org/10.6084/m9.figshare.28271237

**Experimental models: Organisms/strains**		

WT *Danionella Cerebrum*	Benjamin Judkewitz’s laboratory, Charité and Humboldt University, Berlin, DE	N/A

**Software and algorithms**		

Matlab version R2023b	MathWorks	https://it.mathworks.com/products/matlab.html
RStudio version 2023.06.0+421	Posit Software, PBC	https://posit.co/download/rstudio-desktop/
G∗Power version 3.1.9.6	HHU	https://www.psychologie.hhu.de/arbeitsgruppen/allgemeine-psychologie-und-arbeitspsychologie/gpower
GeNEsIS (stimuli creation tool)	Zanon et al.^33^	https://github.com/MirkoZanon/GeNEsIS

### Experimental model and study participant details

Sixteen adult wild-type *Danionella cerebrum* were obtained from Benjamin Judkewitz’s laboratory in Berlin.

The fish were maintained at the CIMeC laboratory in Rovereto in a communal tank (80 liters of recirculating filtered water, supplemented with 20 mL Acquatan and 32 mL Bionitrinex). The tank was kept at an average temperature of 28°C under a 14-hour light/10-hour dark cycle. The fish were fed twice daily with small food pellets or *Artemia*.

The experiments complied with all applicable national and European laws concerning the use of animals in research and were examined and approved by the Ethical Committee of the University of Trento (Organo Preposto al Benessere Animale, OPBA; Protocol n. 06/2024).

Two days before the experiment, the fish were separated into individual compartments corridors within the communal tank, preventing visual contact between the fish and minimizing exposure to external distractions, while maintaining the familiar shared water system. Once immersed, the corridors were filled with water up to a height of 7 cm.

The fish were randomly divided into two experimental groups for a habituation/dishabituation paradigm (details provided below). One group of 8 fish was habituated to a stimulus of 3 dots and dishabituated with 9 dots, while the second group of 8 fish underwent the reverse sequence, being habituated with 9 dots and dishabituated with 3 dots.

### Method details

#### Setup

Two days before the experiment, the fish were separated into individual compartments (corridors) measuring 7 × 7 × 20 cm (width × height × length; Figure 1A) within the communal tank (80 liters of recirculating filtered water, supplemented with 20 mL Acquatan and 32 mL Bionitrinex, average temperature of 28°C under a 14-hour light/10-hour dark cycle). The corridors were constructed using white Poliplack for the sides, and the bottom was covered with a thin mesh net. Once immersed, the corridors were filled with water up to a height of 7 cm. To record the experiment, multiple cameras were positioned 30 cm above the tank to capture the entire setup.

#### Stimuli

The stimuli consisted of two-dimensional square arrays (6 × 6 cm) of dots, featuring either 3 or 9 circular red dots on a white background. The dots were pseudo-randomly arranged to maintain high stimulus variation while balancing and controlling physical variables that could potentially confound numerical estimation, including dot radius, total area, total perimeter, mean inter-dot distance, and convex hull (see some examples in Figure 1B). The smallest element in the whole stimuli set had a radius of 0.2 cm, which was also the minimum elements’ distance possible, ensuring that the visual angle under which fish could observe the dots from the maximum distance of 20 cm was no less than 0.58 deg (corresponding to 1.16 deg per cycle, i.e. 0.86 cpd, above the discrimination thresholds computed e.g. in zebrafish^34^). The stimuli were generated using GeNEsIS.^33^

To ensure the fish habituated solely to numerical information, the continuous confounding geometric variables were systematically varied across trials (see Tables S1–S5) and on average, matched between the two tested numerosities (across the different subjects tested). This approach minimized the influence of non-numerical features on the fish’s responses.

The stimuli were affixed to the bottom section of white Poliplack strips measuring 6.8 × 19.8 cm. These strips were manually inserted along the short side of the experimental corridor, ensuring that the square stimulus was fully submerged in water and clearly visible to the fish.

#### Habituation/dishabituation paradigm

Sample size was evaluated based on alpha level 0.05, with 80% power, and a medium-high effect size (Cohen’s *dz*=0.7 based on previous analogous studies^25^^,^^26^^,^^29^). A matched-paired comparison between two dependent means (considering the average score comparison between habituation day 4 and dishabituation day 5) requires 15 fish. The fish were maintained in individual corridors starting two days before the beginning of the experiment to allow them to familiarize themselves with the new environment. They remained in their respective corridors throughout the entire duration of the experiment.

The habituation/dishabituation paradigm we employed is a well-established method in neuroscience (widely used in infant research, adapted to other animals and more recently to fish^25^^,^^26^^,^^30^^,^^31^^,^^35^), which consists of a prolonged habituation phase followed by a single dishabituation trial. This approach relies on the idea that repeated exposure to a stimulus leads to a reduction in response (habituation), and that presenting a novel stimulus disrupts this condition, allowing researchers to assess discrimination. A single dishabituation trial is sufficient to detect this effect and avoids weakening the novelty response through repetition, while statistical power is achieved by testing multiple individuals rather than repeating dishabituation trials within subjects.

The experiment consisted of a 5-day habituation phase followed by a final dishabituation trial. Each day, the fish were presented with six different stimuli (all of the same numerosity, either 3 or 9 dots) during six consecutive trials lasting 20 minutes each (no pauses were present between trials within a session). Thus, the habituation phase lasted 2 hours per day, with six distinct configurations of the same numerosity presented across trials. The specific configurations varied across days (see Tables S1–S5). On the last (fifth) day, during the final (sixth) trial, a new numerosity was introduced to assess potential dishabituation effects (Figure 1C).

Despite each session was lasting 20 minutes (for habituation porpoises) behavioural data were analysed only during the first few minutes of each session. This approach is consistent with established paradigms in species such as zebrafish,^25^^,^^26^ where behaviour is assessed at the beginning of a trial to avoid confounding effects such as within-trial habituation, fatigue, or loss of interest. Although only a short observation window is used for behavioural analysis, longer exposure periods (i.e., 20 minutes per trial, over six trials across five days) are essential for establishing a robust habituation effect. In this paradigm, extended and repeated exposure allows habituation to develop gradually, while dishabituation is evaluated based on relative changes within and across sessions. Moreover, it’s important to notice that a strong dishabituation effect depends on the perceived novelty of the stimulus. Repeated exposure to the novel stimulus across days would likely diminish its novelty and, consequently, reduce the strength of the behavioural response. Conversely, maintaining the same habituation stimulus across multiple sessions is necessary to ensure that the change introduced in the final trial is meaningful and elicits a clear behavioural shift. Conducting the entire habituation/dishabituation sequence within a single day or multiple times on the same subject (meaning with multiple dishabituation tests) would likely be insufficient to produce a measurable effect. In summary, presenting the dishabituation stimulus only once, after multiple days of consistent habituation, is essential for the validity and effectiveness of the paradigm.

Fish movements and behavior were recorded, and the time spent near the stimulus (within 3 cm close to it - two average body length-) was evaluated as a key metric for constructing the habituation/dishabituation curve.

The fish were tested in the morning, and after the habituation trials concluded (in the afternoon), they were fed with small food pellets. No food was provided during the 2 hours test to avoid any possible distraction or disturbance.

### Quantification and statistical analysis

To construct the habituation/dishabituation curve and evaluate the dishabituation effect, we analyzed the time spent by the fish in proximity to the stimulus (within 3 cm of the short side of the corridor where the stimulus was inserted) during the first 3 minutes of each trial. This time was extracted by visual inspection of the video recording, using a digital chronometer (created with Matlab) to track the seconds spent by the animal close to the stimulus. The timing was measured using a digital stopwatch to progressively save all times spent in different areas, and coding was conducted blindly with respect to experimental condition, twice to ensure reliability. This time was normalized by dividing the seconds spent within the 3 cm proximity region by 180 s. The proportion of time is reported in the habituation/dishabituation curves. The choice of the 3-minute window was motivated by the rationale of allowing the animals some time after stimulus onset, as they could be startled by the stimulus insertion. Nonetheless, scores obtained from different time windows were consistent, as shown in Figure S2.

To evaluate habituation/dishabituation effects, we calculated a differential score for each experimental day as the difference between the proportion of time near the stimulus in the final (sixth) trial and the average proportion of time in the first five trials. This score allowed us to assess whether dishabituation (i.e., a change in time spent near the stimulus) occurred during the final daily trials. The differential score ranges from -2 to 2:

• A score of -2 indicates strong habituation, with the fish spending significantly less time near the stimulus in the final trial (compared to the average of the first trials).
• A score of 0 indicates no change, meaning the fish spent a consistent amount of time near the stimulus across all trials.
• A score of 2 indicates strong dishabituation, where the fish spent much more time near the stimulus in the very final trial.

If numerical dishabituation occurs, we would expect the score to significantly increase on the last day (when the new stimulus is introduced in the final trial).

Note that this score is computed in the exact same way throughout all 5 experimental days. But for clarity, we will refer to it as habituation score while speaking of days 1-4 and dishabituation score while speaking of day 5.

While high scores might also be observed on the first days (as the fish investigate novel stimuli), we would expect the scores to decrease across days 1–4 as habituation progresses, becoming negative. It should instead increase back at day 5 if dishabituation occurs. For this reason, to statistically evaluate habituation, we compared the dishabituation score at day 4 (last full day of habituation) against the zero level using a one sample t-test. A significance with a negative score is indicative of successful habituation. To test the dishabituation effect, we compared the scores between day 4 and day 5, expecting a significant increase in the score from day 4 to day 5 if numerical dishabituation occurred. We performed this last comparison using a permutation ANOVA test (*aovperm*, from *permuco* R package,^36^ with 5000 permutations; this is more robust for small datasets), with ‘*Day’* and ‘*Group type’* as factors. The factor ‘*Group type*’ has two levels: habituation with 3 dots and dishabituation with 9 dots, or habituation with 9 dots and dishabituation with 3 dots. This factor allowed us to investigate whether differences arose during dishabituation, based on whether the fish were habituated to the smallest numerosity and dishabituated with the largest one, or vice versa. In the figures significance (p<0.05) is indicated by an asterisk.

Effect sizes were calculated using eta-squared (η^2^) and partial eta-squared (η_p_^2^) to quantify the proportion of variance explained by each factor in the permutation ANOVA. Eta-squared is calculated as: η^2^ = SS_factor_/SS_total_ , where SS_factor_ is the sum of squares for the factor of interest, and SS_total_ is the total sum of squares in the model. Eta-squared represents the proportion of the total variance explained by the factor. Partial eta-squared is calculated as: η_p_^2^ = SS_factor_/(SS_factor_+SS_error_), where SS_factor_ is the sum of squares for the factor of interest, and SS_error_ is the sum of squares for the residual (error) variance. Partial eta-squared reflects the proportion of the variance explained by the factor relative to the variance not explained by other factors in the model. These effect sizes were interpreted based on conventional thresholds, where η^2^ and η_p_^2^ values around 0.01 indicate small effects, 0.06 medium effects, and 0.14 or higher large effects. By using these metrics, the magnitude of the observed effects was standardized, facilitating comparisons across studies and experimental designs.

To quantify sample size we utilized GPower software.

All statistical analyses were performed in R.
