# Supplementary material for: Numerical discrimination in Danionella
Source: iScience. 2025 Sep 30;28(11):113667. doi: 10.1016/j.isci.2025.113667 (PMC12552912; doi:10.1016/j.isci.2025.113667)
Supplement: Document S1. Figures S1 and S2 and Tables S1–S5 [file mmc1.pdf]

iScience, Volume *28*

Supplemental information

Numerical discrimination in Danionella

Mirko Zanon, Scott E. Fraser, and Giorgio Vallortigara

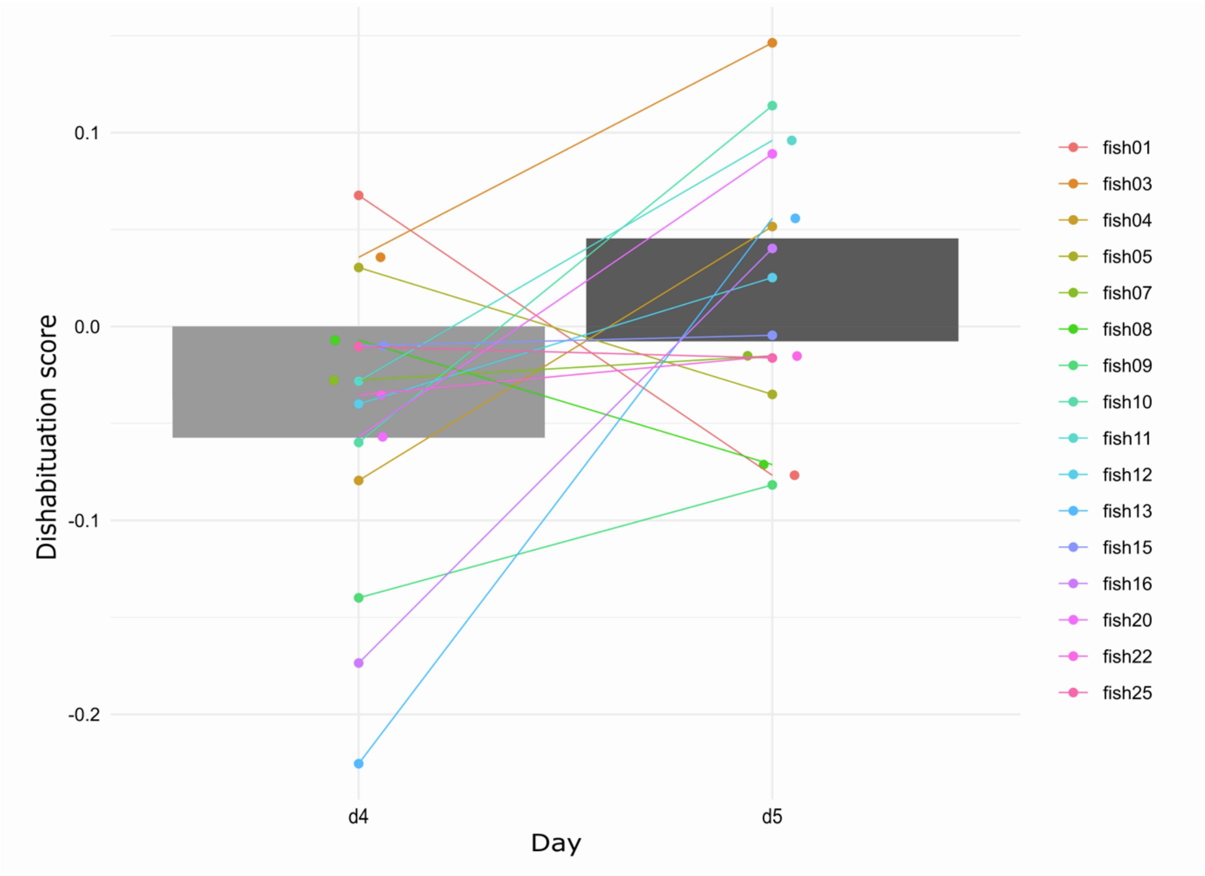

Figure S1. Habituation/dishabituation score per fish.
Differential habituation/dishabituation score (between last trial and previous habituation trials of days 4 and 5), with lineplot for single fish.
Related to main Figure 2b and Figure 3.

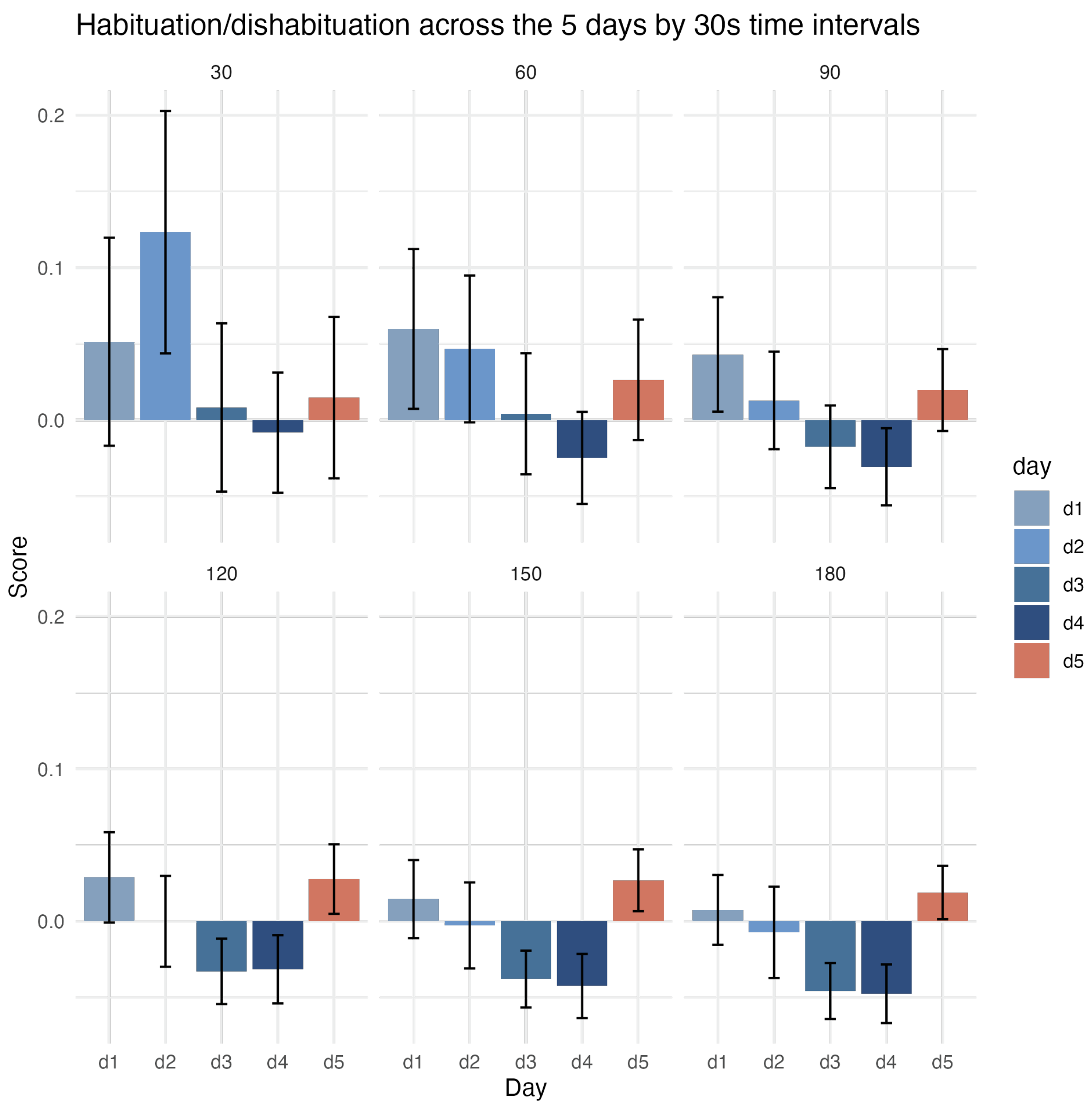

Figure S2. Habituation/dishabituation scores for different time intervals.
Differential dhabituation/dishabituation score (across the 5 days), calculated considering time spent by fish close to stimulus with different time windows from stimulus onset (30 s, 60 s, 90 s, 120 s, 150 s, 180 s).
Related to main Figure 2b.

DAY 1	Trial 1	Trial 2	Trial 3	Trial 4	Trial 5	Trial 6
**Fish 1/9**	CH-R	ID-TA	CH-TP	ID-R	CH-TA	ID-TP
**Fish 10**	ID-TP	ID-R	ID-TA	CH-TA	CH-TP	CH-R
**Fish 3/11/20**	ID-R	ID-TA	CH-TP	CH-R	ID-TP	CH-TA
**Fish 4/12**	CH-TA	CH-TP	ID-TA	ID-TP	CH-R	ID-R
**Fish 5/13/22**	ID-R	CH-TA	ID-TP	CH-R	ID-TA	CH-TP
**Fish 7/15**	ID-TP	CH-R	CH-TA	CH-TP	ID-TA	ID-R
**Fish 8/16/25**	CH-R	ID-R	CH-TA	ID-TP	CH-TP	ID-TA

Table S1. Type of stimuli used for habituation day 1.
Half of the fish were habituated with 3 dots (fish 1/3/4/5/7/8/9/10) and the others with 9 dots (fish 11/12/13/15/16/20/22/25). CH: convex hull at 15.3 cm^2; ID: average inter-dots distance at 3.6 cm^2; R: fix dots' radius at 0.3 cm; TA: dots' total area at 1.8 cm^2; TP: dots' total perimeter at 14.4 cm.
Related to START Methods.

DAY 2	Trial 1	Trial 2	Trial 3	Trial 4	Trial 5	Trial 6
**Fish 1/9**	CH-R	ID-R	CH-TA	ID-TP	CH-TP	ID-TA
**Fish 10**	CH-R	ID-TA	CH-TP	ID-R	CH-TA	ID-TP
**Fish 3/11/20**	ID-TP	ID-R	ID-TA	CH-TA	CH-TP	CH-R
**Fish 4/12**	ID-R	ID-TA	CH-TP	CH-R	ID-TP	CH-TA
**Fish 5/13/22**	CH-TA	CH-TP	ID-TA	ID-TP	CH-R	ID-R
**Fish 7/15**	CH-TA	CH-TP	ID-R	ID-TA	ID-TP	CH-R
**Fish 8/16/25**	ID-TP	CH-R	CH-TA	CH-TP	ID-TA	ID-R

Table S2. Type of stimuli used for habituation day 2.
Half of the fish were habituated with 3 dots (fish 1/3/4/5/7/8/9/10) and the others with 9 dots (fish 11/12/13/15/16/20/22/25). CH: convex hull at 15.3 cm^2; ID: average inter-dots distance at 3.6 cm^2; R: fix dots' radius at 0.3 cm; TA: dots' total area at 1.8 cm^2; TP: dots' total perimeter at 14.4 cm.
Related to START Methods.

DAY 3	Trial 1	Trial 2	Trial 3	Trial 4	Trial 5	Trial 6
**Fish 1/9**	ID-TP	CH-R	CH-TA	CH-TP	ID-TA	ID-R
**Fish 10**	CH-R	ID-R	CH-TA	ID-TP	CH-TP	ID-TA
**Fish 3/11/20**	CH-R	ID-TA	CH-TP	ID-R	CH-TA	ID-TP
**Fish 4/12**	ID-TP	ID-R	ID-TA	CH-TA	CH-TP	CH-R
**Fish 5/13/22**	ID-R	ID-TA	CH-TP	CH-R	ID-TP	CH-TA
**Fish 7/15**	ID-R	CH-TA	ID-TP	CH-R	ID-TA	CH-TP
**Fish 8/16/25**	CH-TA	CH-TP	ID-R	ID-TA	ID-TP	CH-R

Table S3. Type of stimuli used for habituation day 3.
Half of the fish were habituated with 3 dots (fish 1/3/4/5/7/8/9/10) and the others with 9 dots (fish 11/12/13/15/16/20/22/25). CH: convex hull at 15.3 cm^2; ID: average inter-dots distance at 3.6 cm^2; R: fix dots' radius at 0.3 cm; TA: dots' total area at 1.8 cm^2; TP: dots' total perimeter at 14.4 cm.
Related to START Methods.

DAY 4	Trial 1	Trial 2	Trial 3	Trial 4	Trial 5	Trial 6
**Fish 1/9**	CH-TA	CH-TP	ID-R	ID-TA	ID-TP	CH-R
**Fish 10**	ID-TP	CH-R	CH-TA	CH-TP	ID-TA	ID-R
**Fish 3/11/20**	CH-R	ID-R	CH-TA	ID-TP	CH-TP	ID-TA
**Fish 4/12**	CH-R	ID-TA	CH-TP	ID-R	CH-TA	ID-TP
**Fish 5/13/22**	ID-TP	ID-R	ID-TA	CH-TA	CH-TP	CH-R
**Fish 7/15**	CH-TA	CH-TP	ID-TA	ID-TP	CH-R	ID-R
**Fish 8/16/25**	ID-R	CH-TA	ID-TP	CH-R	ID-TA	CH-TP

Table S4. Type of stimuli used for habituation day 4.
Half of the fish were habituated with 3 dots (fish 1/3/4/5/7/8/9/10) and the others with 9 dots (fish 11/12/13/15/16/20/22/25). CH: convex hull at 15.3 cm^2; ID: average inter-dots distance at 3.6 cm^2; R: fix dots' radius at 0.3 cm; TA: dots' total area at 1.8 cm^2; TP: dots' total perimeter at 14.4 cm.
Related to START Methods.

DAY 5	Trial 1	Trial 2	Trial 3	Trial 4	Trial 5	Trial 6 - Test
**Fish 1**	ID-R	CH-TA	ID-TP	CH-R	ID-TA	CH-TP
**Fish 3**	ID-TP	CH-R	CH-TA	CH-TP	ID-TA	ID-R
**Fish 4**	CH-R	ID-R	CH-TA	ID-TP	CH-TP	ID-TA
**Fish 5**	CH-R	ID-TA	CH-TP	ID-R	CH-TA	ID-TP
**Fish 7**	ID-R	ID-TA	CH-TP	CH-R	ID-TP	CH-TA
**Fish 8**	CH-TA	CH-TP	ID-TA	ID-TP	CH-R	ID-R
**Fish 9**	ID-R	CH-TP	ID-TP	CH-R	ID-TA	CH-TA
**Fish 10**	CH-TA	CH-R	ID-R	ID-TA	ID-TP	CH-TP
**Fish 11**	ID-TP	CH-R	CH-TA	CH-TP	ID-R	ID-TA
**Fish 12**	CH-R	ID-R	CH-TA	ID-TA	CH-TP	ID-TP
**Fish 13**	CH-R	ID-TA	CH-TP	ID-TP	CH-TA	ID-R
**Fish 15**	ID-R	CH-TA	CH-TP	CH-R	ID-TP	ID-TA
**Fish 16**	CH-TA	CH-TP	ID-TA	ID-R	CH-R	ID-TP
**Fish 20**	CH-R	ID-TA	CH-TA	ID-TP	CH-TP	ID-R
**Fish 22**	ID-TP	ID-R	ID-TA	CH-R	CH-TP	CH-TA
**Fish 25**	CH-TA	ID-R	ID-TA	ID-TP	CH-R	CH-TP

Table S5. Type of stimuli used for habituation/dishabituation day 5.
Half of the fish were habituated with 3 dots and dishabituated with 9 (fish 1/3/4/5/7/8/9/10) and the others were habituated with 9 dots and dishabituated with 3 (fish 11/12/13/15/16/20/22/25). The dishabituation test occurred in the last trial (Trial 6). CH: convex hull at 15.3 cm^2; ID: average inter-dots distance at 3.6 cm^2; R: fix dots' radius at 0.3 cm; TA: dots' total area at 1.8 cm^2; TP: dots' total perimeter at 14.4 cm.
Related to START Methods.
